# 共价有机框架材料在固相萃取中的最新应用进展

**DOI:** 10.3724/SP.J.1123.2022.12021

**Published:** 2023-07-08

**Authors:** Yiyang GAO, Yali DING, Luyu CHEN, Fang DU, Xubo XIN, Juanjuan FENG, Mingxia SUN, Yang FENG, Min SUN

**Affiliations:** 济南大学化学化工学院,山东济南250022; School of Chemistry and Chemical Engineering, University of Jinan, Jinan 250022, China

**Keywords:** 共价有机框架, 固相萃取, 样品前处理, 有机污染物, 重金属离子, covalent organic frameworks (COFs), solid-phase extraction (SPE), sample pretreatment, organic pollutants, heavy metal ions

## Abstract

固相萃取技术是一种快速简便的样品前处理技术,能够实现分析物的富集,提高分析检测的准确性和灵敏度,已被广泛应用于食品安全检测、环境污染物分析等领域。如何提高方法的灵敏度和选择性是样品前处理领域的不懈追求,其关键在于萃取材料的选择。共价有机框架(COFs)材料是一类晶态多孔聚合物,其首先经热力学控制的可逆聚合反应形成连锁单元,然后与具有一定对称性的有机小分子构筑单元连接而形成,已被广泛应用于气体吸附、催化、传感、药物输送等领域。COFs具有比表面积大、孔隙率高、稳定性好、可设计性强以及合成简单、选择性高等优点,在样品前处理领域中引起了广泛关注。目前,COFs作为一种新型固相萃取材料,已被应用于食品、环境、生物样品中不同类别污染物的萃取富集,如重金属离子、多环芳烃、苯酚、氯酚、氯苯、多溴联苯醚、雌激素、药物残留、农药残留等。采用不同单体合成的COFs对于不同类型分析物具有不同的富集效果,因此也可以通过对其进行修饰合成一种新型COFs,以达到对某种结构分析物的高效选择性富集。本文将介绍COFs的主要类型及合成方法,并重点介绍近3年来COFs作为固相萃取材料在食品、环境、生物领域方面的重要应用,同时展望了COFs在固相萃取领域的发展前景。

## Abstract (en)

Covalent organic frameworks (COFs) are a type of crystalline porous polymers. It firstly prepared by thermodynamically controlled reversible polymerization to obtain chain units and connecting small organic molecular building units with a certain symmetry. These polymers are widely used in gas adsorption, catalysis, sensing, drug delivery, and many other fields. Solid-phase extraction (SPE) is a fast and simple sample pretreatment technology that can enrich analytes and improve the accuracy and sensitivity of analysis and detection; it is extensively employed in food safety detection, environmental pollutant analysis, and several other fields. How to improve the sensitivity, selectivity, and detection limit of the method during sample pretreatment have become a topic of great interest. COFs have recently been applied to sample pretreatment owing to their low skeleton density, large specific surface area, high porosity, good stability, facile design and modification, simple synthesis, and high selectivity. At present, COFs have also attracted extensive attention as new extraction materials in the field of SPE. These materials have been applied to the extraction and enrichment of diverse types of pollutants in food, environmental, and biological samples, such as heavy metal ions, polycyclic aromatic hydrocarbons, phenol, chlorophenol, chlorobenzene, polybrominated diphenyl ethers, estrogen, drug residues, pesticide residues, etc. COFs can be synthesized from different materials and exert different effects on different extracts. New types of COFs can also be synthesized via modification to achieve better extraction effects. In this work, the main types and synthesis methods of COFs are introduced, and the most important applications of COFs in the fields of food, environment and biology in recent years are highlighted. The development prospects of COFs in the field of SPE are also discussed.

在环境、食品、生物等领域的样品分析中往往存在分析物浓度痕量、复杂基质干扰等不利因素,因此在分析过程中必须经过样品前处理来消除或减小样品基质干扰,并同时高效富集目标分析物。样品前处理是整个样品分析过程中最重要的步骤,通常需要大量的时间,且在很大程度上会影响分析效率和结果的准确性。因此,提高样品前处理的效率至关重要。与传统的样品前处理方法相比,固相萃取(solid-phase extraction, SPE)具有耗时短、富集效率高、回收率高、成本低、溶剂消耗少等优点,并且可以与各种分析检测手段相结合,减小了检测误差,提高了分析灵敏度。根据萃取材料的具体形式,SPE技术已经发展了柱固相萃取(CSPE)、分散固相萃取(DSPE)、磁固相萃取(MSPE)、移液枪头固相萃取(PT-SPE)等多种方法,并被广泛应用于食品安全^[1]^、环境检测^[2]^、生物分析^[3]^等领域。SPE的萃取行为主要取决于萃取材料的性能,因此高效SPE材料的发展一直是样品前处理领域的前沿课题。多类高性能的萃取材料被应用于SPE,如介孔材料^[4]^、碳材料^[5,6]^、离子液体材料^[7]^、气凝胶材料^[8]^、金属有机框架材料^[9,10]^等。

共价有机框架(COFs)是一类由轻质元素(如C、H、O、N、B等)通过共价键连接的、具有周期性和良好结晶性的多孔二维或三维有机材料。COFs材料具有比表面积大、孔径大小均匀、密度低、结晶性好、稳定性高、可设计性以及可修饰性等优点,已成为目前功能有机材料化学的研究热点^[11]^。一些COFs已被作为SPE材料用于高效萃取有机污染物^[12]^、农药残留物^[13]^、药物分子^[14]^等。本文总结了COFs的主要类别及其合成方法,并综述了近3年COFs作为萃取材料应用于SPE领域的最新研究进展。

## 1 COFs材料的主要分类

根据共价键的形式,COFs可以分为以下6种类型:(1)含有B-O键的COFs材料;(2)含有C=N键,包括亚胺、腙、三嗪基和吩嗪基等基团的COFs材料;(3)含有C=C键的COFs材料;(4)含有C-N键,包括*β*-酮烯胺、酰亚胺和酰胺等基团的COFs材料;(5)含有B=N键(如环硼氮烷)或N=N键(如偶氮二氧基)的COFs材料。总而言之,应用较广泛的COFs材料主要有3种类型,即硼基COFs、亚氨基COFs和三嗪基COFs。

### 1.1 硼基COFs材料

作为最早开发的一类COFs,硼基COFs具有密度低、比表面积大、热稳定性好(450~600 ℃)等优点。目前所报道的硼基COFs主要有两种类型,一类是通过硼酸自缩合脱水形成硼酸酐键(硼氧六环), Pacholak等^[15]^将三(4-二异丙氧基硼苯基)膦与2,3,6,7,10,11-六羟基三苯醚或2,3,6,7-四羟基-9,10-二甲基蒽进行聚合反应,制备了两种由Lewis碱性中心和Lewis酸性硼原子组成的COFs材料,该类材料表现出一定的结晶度、较高的灵活性和有序不连续性;另一类是由硼酸和酚羟基脱水缩合形成硼酸酯键,Yan等^[16]^以带有酚羟基的COFs为原料,通过N-O双齿配体与三氟化硼乙醚配位,成功合成了该类材料,该材料不仅具有良好的结晶性,还表现出了良好的荧光性能。尽管某些硼基COFs在空气中暴露时仍能够保持较大的孔径和结晶度,但大多数硼基COFs在潮湿空气中或水中不稳定,在某种程度上限制了硼基COFs的实际应用。

### 1.2 亚氨基COFs材料

以亚胺键连接的COFs材料具有许多优异的物理化学性质,是现阶段COFs材料中最热门和应用最广泛的一类,该类COFs材料在许多研究领域表现出了极高的应用价值^[17]^。以亚胺键连接的COFs材料大部分都是通过席夫碱反应合成的。席夫碱反应是指醛或酮与伯胺在特定条件下脱水缩合形成碳氮双键(-C=N-)的反应,反应条件温和,反应过程受热力学控制且产品的产率和结晶度均较高。与硼基COFs相比,亚氨基COFs的稳定性更好,这为亚氨基COFs材料的实际应用提供了更多的可能性。2009年,Uribe等^[18]^最早合成出亚氨基COFs,以四(4-氨基苯基)甲烷与对苯二甲醛为有机单体进行缩聚反应,得到了由亚胺键连接的、具有高热稳定性(490 ℃)与良好化学稳定性的三维COFs(COF-300),且该三维COFs具有较大的比表面积(1360 m^2^/g)。随后该课题组用酰胺、酰肼和醛基反应生成腙键连接的高结晶COF-42和COF-43^[19]^,由于腙的动态可逆缩合构造增强了其活化性能,两种材料均表现出优异的化学稳定性和热稳定性。这些坚固的COFs分子中含有较多的活性官能团,在孔隙之间引入了新的极性和氢键,促进了对COFs性能的探索。近年来,亚胺类COFs已被广泛应用在各个领域,如食品^[20]^、环境^[21]^、生物^[22]^、催化^[23,24]^等。

### 1.3 三嗪基COFs材料

三嗪共价有机骨架(CTFs)材料是由芳香腈化合物经环化反应制备而成的多孔材料,具有良好的化学稳定性和热稳定性。CTFs的显著优势是其结构中含有三嗪基,使材料表面具有一定的极性,且该材料具有可调节的表面积和孔隙率。传统的CTFs材料一般通过离子热或强酸催化等方法制备,反应条件苛刻,单体来源有限。Kuhn等^[25]^在2008年利用对苯二腈在氯化锌的催化下通过离子热反应制备了一种二维层状材料CTF-1,这是被报道的首个三嗪类COFs,其孔隙率高,比表面积大,性能与沸石、共价氧化硼基骨架和金属有机骨架材料相当。2020年,Zhao等^[26]^通过离子热法合成了一种磺胺基功能化三嗪类COFs(CTF-SO_3_H),其孔隙高度开放,比表面积大,稳定性好。此外,Yang等^[27]^采用离子热法制备了S基团修饰的CTFs,合成的材料对过渡金属离子尤其是Hg^2+^具有高选择性,在较宽的pH范围内具有高稳定性和良好的回收率,连续5个循环中,对Hg^2+^的去除率高达94%。近年来,相比于亚氨基COFs的广泛应用,CTFs的应用有限。

## 2 COFs材料的主要合成方法

COFs的合成方法主要有溶剂热法、离子热法、微波合成法和室温合成法。其中,溶剂热法是最常用的合成方法; 离子热法是一种新型的合成方法; 与微波合成法相比,溶剂热法更加节能; 室温合成法是一种绿色的制备方法。此外,还有光化学合成法等其他合成方法。

### 2.1 溶剂热法

溶剂热法是指在密闭体系内(如高压釜),以有机物或非水溶媒为溶剂,在一定温度和压力下进行反应的一种合成方法,是一种最常用的COFs合成方法。Hu课题组^[28]^采用溶剂热法,将2,4,6-三羟基苯-1,3,5-三乙醛、4,4-二氨基联苯和4-氨基苯硼酸混合,在80 ℃下反应4 h合成了一种新型的高选择性吸附材料B-COFs。结果表明,B-COFs与其他传统材料相比具有显著优势,如比表面积大、硼酸位点多、稳定性高、选择性富集能力强、适用性好,其对顺式二醇化合物具有较好的选择性,也是一种萃取和富集顺式二醇化合物的新型材料。Xiong等^[29]^采用溶剂热法,用三(4-氨基苯基)胺(TAPA)和三(4-甲酰基苯基)胺(TFPA)合成了席夫碱型TATF-COFs。在发生溶剂热反应的过程中,动态亚胺键的可逆反应使COFs从纳米粒子形态转变为纳米纤维。TATF-COFs具有较高的着色效率、中等的对比度和切换速度,可应用于制造高性能电致变色材料。Li等^[30]^首次构建了Zr^4+^固定化共价有机框架(Fe_3_O_4_@COF@Zr^4+^),并将其作为磁性固相萃取有机磷农药(OPPs)的高效吸附剂。通过*π-π*堆积和氢键作用,该复合材料对OPPs表现出优异的选择性和亲和力,回收率为87%~121%,相对标准偏差(RSD)为5%~8.9%。这些结果表明,所制备的纳米颗粒在食品OPPs的痕量检测中具有独特优势。但传统溶剂热法存在一些局限性,如反应时间长、反应温度高、反应效率低,因此开发一种能够提供更好的反应效率和空间可控性的新型合成方法已成为一个关键挑战。

### 2.2 离子热法

离子热法是一种以离子液体或低共熔混合物为介质的新型分子筛合成方法,其仅用于CTFs的合成。CTFs多为非晶态材料,在离子热条件下可制备出具有结晶性的CTFs,该方法合成的CTFs材料孔隙多,稳定性高,表面积大,但合成过程耗费大量的时间。2008年,Kuhn等^[25]^通过离子热法合成了多孔晶体CTF-1,其具体合成步骤如下:将单体1,4-二氰基苯和金属盐氯化锌加入安瓿瓶中,然后将安瓿瓶抽真空、封口并在400 ℃下反应40 h; 待体系温度降至室温后打开安瓿瓶,所得固体即为CTF-1。在反应过程中,熔融ZnCl_2_起着至关重要的作用,它不仅是反应溶剂,而且是可逆氰基三聚化反应的催化剂。Chen等^[31]^采用离子热法成功合成了4种具有高比表面积的CTFs材料,该方法通过调节温度来调控所得产物的孔径和比表面积。在N_2_气氛下以无水ZnCl_2_为催化剂和反应介质,在400~500 ℃下通过离子化反应合成了基于两种芘连接剂的新型CTFs材料,且产率超过70%,将其应用于混合气体的分离,取得了较为满意的结果。

### 2.3 微波合成法

微波合成法是指利用微波加热的合成方法,与溶剂热法相比,它具有快速、高效、节能等优点,在快速筛选最优反应条件和提高反应速率等方面具有较大优势,极大地促进了COFs材料的发展。Chen等^[32]^通过微波辅助溶剂热方法,以1,3,5-三(4-氨基苯基)苯(TAPB)和2,3,5,6-四氟邻苯二甲醛(TFA)或1,4-邻苯二甲醛(PDA)为单体合成COFs,苯环上F基团的引入提升了COFs的疏水性和稳定性,在微波下加热反应1 h得到TAPB-TFA-COF和TAPB-PDA-COF,其产率达80%~85%。COFs作为新型吸附剂,表现出优异的油包水乳状液分离性能,可实现普遍且长效的吸附油过程,分离效率不低于99.5%。Ding等^[33]^基于快速微波辅助阴离子交换反应,提出了一种新型的阳离子COFs孔道壁修饰策略。先以溴化乙啶(EB)和1,3,5-三甲酰基间苯三酚为原料,以1,4-二氧六烷、均三甲苯、乙酸为溶剂介质,在微波合成器中于100 ℃加热3 h合成了一种带有溴离子的COFs,进而通过微波辅助阴离子交换反应发展了3种其他阴离子的COFs材料。Guntern等^[34]^以1,3,5-间苯三甲醛(TFB)、4-(叔丁氧酰胺基)苯胺(NBPDA)、聚乙烯吡咯烷酮、纳米晶(NC)和三氟乙酸为原料,以乙醇为溶剂,将反应物混匀后转移到微波玻璃瓶中,在5 min内加热到120 ℃,保持30 min,然后冷却至室温,将以均三苯甲醛和对苯二甲胺为单体所合成的COFs(LZU1)去质子化,得到了核壳型的NC@COF-LZU1材料。该研究提出了一种新的功能化COFs思路,即结合胶体力学和微波合成方法,在NC存在时形成良好结晶度的LZU1, LZU1壳层的厚度能够被有效调控,并且该方法对于合成NC/COFs杂化材料具有良好的通用性。

### 2.4 室温合成法

大多数COFs材料是通过溶剂热法合成的,但室温合成法由于其绿色、简单、易操作和成本低等优点,已逐渐成为一种热点。Liu等^[35]^通过在室温下以4,4,4-(1,3,5-三氮嗪2,4,6-三基)三苯胺(Tz)和1,4-二羟基对苯二甲醛(Da)为单体合成COFs(TzDa),并引入手性选择剂,设计合成了手性COFs(CTzDa)。所制备的CTzDa在各种溶剂中均表现出良好的稳定性,对氨基酸对映体(L-AAs与D-AAs)的吸附分离速度快、选择性高。结果表明,L-AAs与D-AAs之间立体氢键的差异对CTzDa的选择性吸附起到了关键作用。该研究为氨基酸对映体的高选择性分离提供了一种简便的策略,也展示了CTzDa在手性氨基酸色谱分离中的潜力。Medina等^[36]^在室温下通过蒸汽辅助转化的简便方法合成了几种二维COFs。在不同的条件、不同的基底上合成了苯并二噻吩基COFs(BDT-COF)和COF-5薄膜。微米级的BDT-COF薄膜表现出介孔性和纹理孔隙,而较薄的BDT-COF薄膜物化为内聚致密层。该研究表明,对于在溶剂热反应条件中的敏感单体和敏感基底上生成COFs膜,室温蒸汽辅助转化法是一种理想的方法。

### 2.5 其他合成方法

除上述4种主要合成方法外,还有一些其他合成方法。例如,Kim等^[37]^首次提出了用光化学合成法制备C_9_H_4_BO_2_(UV-COF-5),产物为尺寸均匀的“海胆状”结构,合成速率远快于溶剂热法,且产率理想(75%)。由于其分层形态,UV-COF-5的比表面积大(2026.9 m^2^/g); 其次,它的制备方法简单,无需任何复杂合成后的光刻工艺,只需在光化学方法中使用光学掩模即可。此外,界面聚合法也已被广泛用于合成COFs, Zhang等^[38]^采用界面聚合法和层层堆积的工艺将EB和1,3,5-三甲酰氯丁二醇(TFP)通过席夫碱反应合成了稳定的酮烯醇互变异构基EB-COF膜。

## 3 COFs在固相萃取领域的应用

### 3.1 COFs在食品领域的应用

随着人们生活水平的提高,食品安全问题越来越引起人们的重视。农药的滥用、饲料添加剂的过度使用等给人民群众的健康带来了一定的危害,人们对食品安全检测的要求越来越高。因此,各种高效的前处理材料被应用于食品样品的预处理,COFs作为一类高性能的萃取材料在食品领域的应用也越来越广泛。附表1(www.chrom-China.com)中列举了一些COFs基材料在食品、环境、生物领域中的应用。

针对农药的滥用,Zhao等^[26]^采用离子热法以1,4-二氰苯为原料合成了负载镍纳米颗粒的亚磺酸功能化共价三嗪有机框架(Ni/CTF-SO_3_H),将其作为MSPE材料用于萃取蔬菜、水果和果汁中的苯并咪唑杀菌剂(多菌灵、噻菌灵),并用高效液相色谱-紫外检测器(HPLC-UV)检测。此方法萃取时间短,灵敏度好,准确度高,检出限(LOD)为1.23~7.05 μg/kg,线性范围宽至7.90~1000 μg/kg,回收率为80.2%~115.1%,且RSD值较小(6.0%~11.4%),说明该方法具有良好的实用性和准确性,然而其灵敏度相对较低。为此,Xu等^[39]^采用室温合成法,在羧基化四氧化三铁纳米颗粒表面用1,3,5-三(4-氨基苯基)三嗪(TAPT)和2,3,5,6-四氟对苯二醛(TFTA)原位聚合成功合成了一种具有良好磁响应性和丰富氟亲和位点的核壳结构磁性COFs(Fe_3_O_4_@TAPT-TFTA-COF)。将MSPE与高效液相色谱-串联质谱(HPLC-MS/MS)相结合建立分析方法并用于黄酒和果汁样品中苯甲酰脲杀虫剂的检测。实验结果显示其线性检测范围分别为10~2000 ng/L和20~4000 ng/L, LOD低至0.4 ng/L,定量限(LOQ)为1.4~13.3 ng/L, RSD低于11%。

Xu等^[20]^以1,3,5-三(4-氨基苯基)和2,5-二羟基对苯二甲醛(DHTA)为单体,采用简易室温法制备了三嗪类COFs(TAPT-DHTA-COF),将其作为SPE材料,并采用HPLC-MS/MS对水稻、苹果和蔬菜样品中的一类重要农药残留苯氧羧酸(PCA)进行检测。实验得到的线性范围为0.10~40 ng/g, LOD为0.007~0.030 ng/g,实际样品中PCA的加标回收率为81.2%~107%。结果表明,该方法在PCA残留分析中具有较高的灵敏度和准确性,有良好的应用潜力。此外,Gao等^[13]^提出了一种原位合成修饰策略,以三维石墨烯基复合材料(3DGA)为载体,制备了一种1,3,5-苯三甲醛和联苯胺(BD)为单体的COFs复合材料(3DGA@COFs),将其作为SPE材料用于蔬菜中OPPs的萃取和富集。制备的3DGA@COFs材料具有大比表面积(127.95 m^2^/g)、高孔隙率和大孔体积(0.0344 cm^3^/g)等优点,大大提高了萃取效率。将其与HPLC-MS/MS分析系统联用,结果表明,该方法线性范围宽(0.5~100 μg/L), LOD低(0.01~0.14 μg/L),回收率较高(75.40%~102.13%),并成功用于生菜、番茄、黄瓜样品中马拉硫磷、三唑磷、喹啉磷的测定,表明3DGA@COFs材料对蔬菜样品的前处理具有应用潜力。

低浓度植物生长调节剂对人体几乎无危害,而长时间的蓄积则会对人体产生不利影响。Li等^[40]^以四氧化三铁纳米颗粒为磁核、三醛基间苯三酚(Tp)和2,6-二氨基蒽醌(DA)为单体,合成了核壳结构的Fe_3_O_4_@TpDA-COF,并将其应用于果蔬中植物生长调节剂(PGRs)的MSPE。该萃取材料具有大的比表面积和高饱和磁化强度以及良好的萃取性能,这些特点使其成为样品预处理的理想萃取材料。基于该材料所发展的分析方法中,LOD为4.68~7.51 μg/L。为了提高分析方法的灵敏度,Tian等^[41]^通过溶剂热法将一种COFs网络(三聚氰胺和间苯二甲醛合成的新型COFs, SNW)与聚丙烯腈(PAN)结合制备了一种新型COFs基萃取材料(SNW-COF-PAN),通过PT-SPE联用高效液相色谱-二极管阵列检测器(HPLC-DAD),快速、灵敏地检测了西瓜中的10种PGRs。所建立的方法线性范围为5~500 ng/mL,相关系数*R*≥0.9981, LOD为0.24~3.19 ng/mL, LOQ为1.65~5.72 ng/mL,日内精密度(≤2.7%)和日间精密度(≤3.7%)良好,加标回收率为82.8%~113.0%。

Wang等^[42]^以Tp和对甲苯磺酸(Tt)合成了一种新型的富氮共价有机框架(TpTt-COFs),将其作为一种新型SPE材料用于萃取饮用水中4种超微量水质消毒副产物(DBPs),采用气相色谱-串联质谱法(GC-MS/MS)进行定量检测。结果表明,该方法的LOD为0.4~6.3 ng/L,线性范围为0.002~50 μg/L, LOQ为1.2~20.8ng/L,且具有良好的重现性。同年,Zhang等^[43]^通过溶剂热法制备了三叶草状纳米钛功能化COFs(CSTF-COFs),羟基的存在使CSTF-COFs可以通过氢键、疏水和亲水作用、*π-π*作用对极性范围较广的*N*-亚硝胺有较高的萃取效率。将CSTF-COFs作为DSPE萃取材料与LC-MS/MS结合,用于测定饮用水中8种*N*-亚硝胺。实验结果表明,该方法对饮用水中*N*-亚硝胺具有较好的萃取性能,LOD为0.13~2.45 ng/L,线性范围为0.5~50.0 ng/L。此方法的LOD显著低于大部分现有方法,CSTF-COFs有望成为一种对饮用水样品中其他污染物监测的高效SPE材料。

Yang等^[44]^制备了*β*-环糊精功能化(Au-*β*-CD)的磁性COFs(Fe_3_O_4_@COF@Au-*β*-CD),不仅保留了COFs比表面积大、孔隙率高的特性,还具有*β*-CD赋予的识别功能,增强了对目标的富集能力。将其作为MSPE材料并与HPLC-MS/MS联用,测定肉类样品中的痕量磺胺类化合物(SAs), LOD为0.8~1.6 μg/kg,线性范围为2~100 μg/kg,相关系数*R*≥0.9936,回收率较高。为增加检测样本的种类,Chen等^[45]^制备了一种与上述相似的磁性纳米复合材料,将COFs修饰在磁性MOF表面,形成一个磁性纳米球(MOF@COF杂化磁性纳米球)并用作MSPE材料,再采用HPLC测定肉类与饮用水中的6种SAs。与上述方法相比,该方法具有更宽的线性范围(10~2000 ng/mL),更高的精密度和准确度。并且该方法只需少量的萃取材料(10 mg)即可获得较高的萃取性能,表明MOF层和COF层均具有良好的萃取性能。此外,Wang等^[46]^采用2,2-二噻吩-5,5-二羧醛和三聚氰胺为单体进行羟醛缩合反应,制备了一种新型CTF作为萃取材料用于肉类中14种SAs的萃取,并结合超高效液相色谱-四极杆-飞行时间质谱仪(UHPLC-Q/TOF-MS)进行检测。该方法的LOD为0.05~0.54 ng/g,线性范围为2.5~100 ng/g,回收率为84.1%~91.9%, RSD为3.2%~4.8%,实验结果表明该方法对复杂样品中SAs的测定具有良好的适用性。在上述3种方法的比较中,COFs用于SAs的检测有望向着LOD更低同时线性范围更宽的方向发展。

Wang等^[47]^用1,3,5-三(4-氨基苯基)-苯(TAPB)和Tp制备了一种具有核壳结构的磁性共价有机框架纳米复合材料(Fe_3_O_4_@TAPB-Tp),建立了磁固相萃取-超高效液相色谱-串联质谱(MSPE-UHPLC-MS/MS)方法检测牛奶、玉米和鸡蛋样品中的5种玉米赤霉烯酮及其衍生物(ZEAs)。该方法的LOD为0.003~0.018 μg/L,线性范围为0.1~100 μg/kg, RSD为2.37%~10.4%,加标回收率为81.27%~90.26%。结果表明,Fe_3_O_4_@TAPB-Tp对于食物中ZEAs的吸附具有良好的潜力。Hong等^[48]^采用溶剂热法以2,4,6-三羟基苯-1,3,5-三羧醛和2,4,6-三(4-氨基苯基)-1,3,5-三嗪(TAPT)为原料合成了一种亚氨基COFs,即TAPT-AN-COF,将其应用于SPE并与HPLC-UV联用测定牛奶和肉类样品中的硝基咪唑药物(NDZs),该方法的LOD为2~10 μg/kg,线性范围为25~500 μg/kg, RSD小于15.9%。

Ma等^[49]^利用Tp中的醛基和BD中的氨基进行缩合反应,并与树脂结合制备了新型经济的COFs(TpBD-COF),将其作为SPE材料检测饮料中4种酚类内分泌干扰物。该方法的RSD小于3%,回收率为98.18%~102.18%,为COFs作为SPE填料用于饮料样品中微量有害物质的HPLC测定提供了新的途径,也为其工业生产提供了可能。此外,Jiang等^[50]^利用TAPB、磁性改性锌基MOF(ZIF-8)、对苯二甲酸(TPA)制备了一种由MOFs和COFs组成的多孔复合材料(Fe_3_O_4_@TAPB-COF@ZIF-8),并将其作为新型MSPE材料与HPLC结合对功能饮料中的微量双酚类污染物进行分析检测。该分析方法的LOD为0.04~0.05 ng/mL, LOQ为0.13~0.15 ng/mL,线性范围较宽(0.25~1000 ng/mL),对4种功能饮料的加标回收率为66.2%~116.6%。说明该方法在功能饮料的微量双酚污染物分析中具有良好的应用价值,在其他食品样品的分析中具有潜在的应用价值。

制备尺寸均匀的COFs是一个巨大的挑战。Li等^[51]^通过室温合成法制备了花型结构的COFs,然后利用表面分子印迹反应合成了一种尺寸均匀的COFs纳米花(COF@MIP),将其作为SPE材料,利用HPLC对谷物中的曲霉素(ST)进行选择性测定。实验结果显示,其LOD为0.001~1.94 μg/L,线性范围为1~1000 μg/L,实际样品中5种谷物的加标回收率为78.6%~98.5%,该方法具有良好的应用前景。

### 3.2 COFs在环境领域的应用

随着环保观念的深入人心,人们对环境污染的关注度逐渐增加。COFs具有结晶性较高、稳定性较好、可设计性以及可修饰性等优点,因此其在环境样品前处理中的应用增多。

Tan等^[52]^用1,3,5-三甲酰氯葡萄糖醇和4,4-乙二胺(EDA)合成了巯基功能化分层多孔COFs(HP-TpEDA-SH),并将其作为SPE材料联合HPLC-UV检测渔业水中痕量阳离子染料亚甲基蓝、孔雀绿、结晶紫(MB、MG、CV)。在最佳萃取条件下,LOD分别为1.3 μg/L(MB)、0.13 μg/L(MG)、0.12 μg/L(CV),线性范围分别为5~500 μg/L(MB)、0.5~50 μg/L(MG)、0.5~500 μg/L(CV), 3个加标水平下的回收率为81.5%~113.8%, RSD均小于9.7%。

与Fe_3_O_4_@COF相关的材料也被广泛应用于环境水样中污染物的检测。Fu等^[53]^在室温条件下利用1,3,5-三(4-氨基苯基)苯和1,4-二醛基-2,5-二乙烯基苯为单体合成了COFs(COF-V),并将COF-V与硅胶和磁性Fe_3_O_4_结合,合成了新型核壳结构的Fe_3_O_4_@SiO_2_@COF-V。将其与气相色谱-负化学电离源质谱(GC-NCI/MS)联用,首次应用于MSPE富集水样中的痕量多溴联苯醚(PBDEs)。结果表明,该方法富集因子高(275~292), LOD低(0.12~0.38 ng/L),线性范围为0.5~1000 ng/L,重现性好,成功检测了不同水样中的PBDEs。Bi等^[54]^合成了硫醇和硫醚功能化磁性COFs(Fe_3_O_4_@COF-S-SH),并通过MSPE和高效液相色谱-电感耦合等离子体质谱(HPLC-ICP-MS)测定化合物中的痕量汞元素,包括无机汞(IHg)、甲基汞(MeHg)和乙基汞(EtHg)。该方法的LOD低至0.17 ng/L,线性范围均良好,对200 mL水样中IHg、MeHg、EtHg的实际富集倍数分别为370、395、365,在实际水样中的加标回收率为96%~108%。该方法灵敏度高,已成功应用于水和鱼类等样品中痕量汞形态的分析。Wang等^[55]^在室温下制备了一种基于NiFe_2_O_4_的磁性共价有机框架纳米复合材料(NiFe_2_O_4_@COFs),其具有较大的比表面积、均匀的孔径、良好的化学稳定性和热稳定性。该研究建立了一种水体中溴化阻燃剂(BFRs)的MSPE-HPLC-UV检测方法。由于该纳米复合材料与BFRs之间的范德华力、疏水作用和*π-π*作用较强,在最佳条件下,该方法的LOD为0.03~1.9 μg/L,线性范围为0.11~1000 μg/L,加标回收率为91.5%~102%, RSD为2.9%。实验结果表明,NiFe_2_O_4_@COFs对BFRs具有良好的吸附性能,在环境应用方面有巨大的潜能。Li等^[56]^采用加热回流法以Tp和BD制备了核壳型Fe_3_O_4_@TpBD-COF材料,对水样中新出现的DBPs进行MSPE前处理,并采用UHPLC-MS/MS分析方法进行检测,LOD为0.07~1.81 ng/L, LOQ为0.24~5.99 ng/L,线性范围为0.5~500 ng/L,二级出水中DBPs的回收率为86.8%~115.1%。该方法是一种快速、高效测定DBPs的样品分析方法。Cao等^[57]^通过室温合成法以TAPB和TPA为单体制备了核壳结构的磁性COFs(Fe_3_O_4_@TaTp-COF),并用作海水和贝类中冈田酸(OA)的MSPE材料,结合LC-MS/MS进行检测。该方法的线性范围分别为5~2000 pg/mL(海水)和0.4~40 μg/kg(贝类),海水的LOD为0.5 pg/mL,贝类的LOD为0.04 μg/kg。与之前的报道相比,提取分析物所使用的材料量减少,所需的时间大大缩短。实验结果表明该方法适用于样品中OA的灵敏检测。

Romero等^[58]^用1,3,5-三磷酰间苯三酚和邻甲苯胺(BD-Me)合成了TpBD-Me_2_ COFs并采用超声辅助分散微固相萃取(US-D-μSPE)方法来萃取海水中的1,3,4,6,7,8-六氢-4,6,6,6,8-六甲基环五-2-苯并吡喃(HHCB)和7-乙酰-1,1,3,4,4,6-六甲基四氢(AHTN)两种多环合成香料。两种分析物的萃取效率为91.2%~97.8%, LOD分别为0.070 μg/L和0.082 μg/L。该方法成功用于海水中多环类有机污染物分析,加标回收率为90.4%~101.2%, RSD为5.8%。基于该材料的分析方法有助于提高选择性,可以减少预处理步骤和色谱分析,在环境中污染物的检测和控制方面具有良好的应用前景。

### 3.3 COFs在生物领域的应用

随着科学技术的发展,社会对生物分析的精度要求提高,急需新工艺、新材料的支撑,并且生物样品成分复杂,部分成分含量低,检测难度高,COFs可满足当前需求,在生物分析领域得到了良好的应用。

Wang等^[59]^用机械搅拌法制备了含氨基核壳结构的COFs纳米球(Fe_3_O_4_@TpBD(NH_2_)_2_),用2-甲酰苯硼酸对Fe_3_O_4_@TpBD(NH_2_)_2_进行修饰,合成了一种新型的磁性硼酸盐亲和材料(Fe_3_O_4_@COF@2-FPBA)。该材料具有快速的磁性响应,对多巴胺具有强结合能力,高达1037 mmol/g。将该材料作为MSPE材料,与高效液相色谱-荧光检测法(HPLC-FLD)联用检测尿单胺神经递质(MNTs)。在最优条件下,线性范围为2~200 ng/mL, LOD为0.31~0.54 ng/mL, LOQ为1.04~1.80 ng/mL,回收率为86.3%~115%。利用这些优点,将纳米复合材料应用于中性条件,从尿液样品中富集5种MNTs,建立了一种检测人尿液中MNTs的高灵敏度方法。该方法具有良好的精密度并成功应用于尿样的测定,对之后的样品前处理工作具有重要意义。

2022年Yu等^[60]^用2,4,6-三羟基苯-1,3,5-三碳醛和对苯二胺(Pa-1)制备了一种用于MSPE技术的磁性COFs(TpPa-1)复合材料,其合成过程与磁性COFs材料相似,只是添加了磁性NH_2_-Fe_3_O_4_纳米颗粒,并用HPLC-MS/MS分析尿液中的芳香胺代谢物(AAs)(1-萘胺、2-萘胺、3-氨基联苯和4-氨基联苯)。4种AAs的LOD和LOQ分别为0.01~0.07 ng/mL和0.04~0.22 ng/mL,加标回收率为81.9%~120%,日内精密度良好。近几年的研究表明COFs在人类体液样品前处理中具有良好的应用前景。

Cao等^[61]^使用溶剂热法制备了磁性Fe_3_O_4_@TpBD-COF,将其作为MSPE材料,并与UHPLC-MS/MS联用对大鼠血浆中的微量安格鲁苷C(Ang C)进行检测。在优化的条件下,线性范围为0.1~5 ng/mL,方法准确度好。由于Fe_3_O_4_@TpBD-COF的比表面积大(110.9 m^2^/g)、磁性响应度高(35.67 emu/g),其萃取效率高且分离方便。未来COFs在生物领域应朝着灵敏度高、操作简单、分析时间短的方向发展,并继续探索同时测定更多目标成分的方法。

## 4 总结与展望

综上,COFs作为新型萃取材料在固相萃取领域已经展现出了良好的应用前景。近年来,由于COFs具有密度低、可修饰性、高比表面积及高孔隙率等优点,使其对各类物质展示出优异的萃取效率,在分析应用中引起了广泛关注。COFs及其复合材料作为萃取材料可以用于各种固相萃取方法,如SPE、DSPE、MSPE和CSPE等,其中多数用于MSPE。目前,COFs及其复合材料在有毒有害物质分析和生物样品前处理中已成为一种优良的萃取剂,与其他报道的商业萃取剂相比,COFs具有更加优异的萃取性能。目前,COFs及其复合材料在样品制备方法中的研究仍处于不断发展中,仅有少数COFs材料应用在固相萃取领域,更多性能优异的COFs材料还有待发掘。COFs合成方法普遍较复杂,成本高,条件苛刻,费时,部分原料有毒性,因此绿色环保的室温合成法有待进一步探索。未来需要制备高选择性、绿色环保、成本低的COFs,推进COFs的商业化,并发展自动化的分析检测方法。随着研究人员的不断探索,会有更多富集效率高的新型COFs材料出现,其所适用的分析物质和应用范围也会更加广泛。
